# Genomic insights into phage-host interaction in the deep-sea chemolithoautotrophic *Campylobacterota*, *Nitratiruptor*

**DOI:** 10.1038/s43705-022-00194-5

**Published:** 2022-11-01

**Authors:** Yukari Yoshida-Takashima, Yoshihiro Takaki, Mitsuhiro Yoshida, Yi Zhang, Takuro Nunoura, Ken Takai

**Affiliations:** 1 Super-cutting-edge Grand and Advanced Research (SUGAR) Program, Institute for Extra-cutting-edge Science and Technology Avant-garde Research (X-star), Japan Agency for Marine-Earth Science and Technology (JAMSTEC), Yokosuka, Kanagawa Japan; 2 Deep-Sea Bioresource Research Group, Research Center for Bioscience and Nanoscience (CeBN), Japan Agency for Marine-Earth Science and Technology (JAMSTEC), Yokosuka, Kanagawa Japan

**Keywords:** Microbial ecology, Bacteriophages, Water microbiology

## Abstract

The genus *Nitratiruptor* represents one of the most numerically abundant chemolithoautotrophic *Campylobacterota* populations in the mixing zones of habitats between hydrothermal fluids and ambient seawater in deep-sea hydrothermal environments. We isolated and characterized four novel temperate phages (NrS-2, NrS-3, NrS-4, and NrS-5) having a siphoviral morphology, infecting *Nitratiruptor* strains from the Hatoma Knoll hydrothermal field in the southern-Okinawa Trough, Japan, and conducted comparative genomic analyses among *Nitratiruptor* strains and their phages. The *Nitratiruptor* temperate phages shared many potential core genes (e.g., integrase, Cro, two structural proteins, lysozyme, and MazG) with each other despite their diverse morphological and genetic features. Some homologs of coding sequences (CDSs) of the temperate phages were dispersed throughout the non-prophage regions of the *Nitratiruptor* genomes. In addition, several regions of the phage genome sequences matched to spacer sequences within clustered regularly interspaced short palindromic repeats (CRISPR) in *Nitratiruptor* genomes. Moreover, a restriction-modification system found in a temperate phage affected an epigenetic feature of its host. These results strongly suggested a coevolution of temperate phages and their host genomes via the acquisition of temperate phages, the CRISPR systems, the nucleotide substitution, and the epigenetic regulation during multiple phage infections in the deep-sea environments.

## Introduction

Deep-sea hydrothermal vents are characterized by high hydrostatic pressure, complete darkness, and steep chemical and physical gradients resulting from the mixing of reductive hot hydrothermal fluids and oxidative cold deep-seawater. Phylogenetically highly diverse microorganisms occupy the diverse ecological niches in the vicinity of vents, contrasting sharply with the surrounding sparsely populated environments in the deep-sea [1–5]. The primary production in the deep-sea vent ecosystem is sustained by chemolithoautotrophic microorganisms that utilize reduced chemical compounds from the earth’s interior as electron donors [6, 7].

*Campylobacterota* (formerly Class *Epsilonproteobacteria*) is a predominant bacterial primary producer in hydrothermal mixing zones [3, 8, 9]. Among the deep-sea vent *Campylobacterota* genera including both free-living forms and epi- or endosymbionts of invertebrates, intraspecific variability in energy metabolism along with available redox couples is observed [3, 10]. Nakagawa et al. indicated that the deep-sea vent *Campylobacterota* diverged from a common ancestor of this lineage before an occurrence of their pathogenic members, such as *Helicobacter* and *Campylobacter* species [10]. Several studies also revealed great plasticity of their genome structures and gene repertoire that could be caused by a high frequency of recombination, mutation, gene loss, or horizontal gene transfer [11–14].

Inducible prophages are frequently found in the genomes of marine bacteria [15, 16]. Phages affect microbial genome diversification and evolution by horizontal gene transfer and lysogenic conversion, which could result in increased fitness of their host microbes in a specific ecological niche [17]. Most pathogenic *Campylobacter* isolates have one or more prophages known as *Campylobacter jejuni* integrated elements (CJIEs) [18–20]. CJIEs are widely distributed among *C. jejuni* strains isolated from various clinical and veterinary sources in many geographical locations [21–24]. The distribution pattern of CJIE sequences is variable at the strain level, and some strains lack CJIE. These results point to a possibility of genomic diversity and differentiation among *C. jejuni* strains driven by highly diverse CJIEs. Furthermore, CJIEs can affect the physiological properties and virulence of the host bacterium. For example, the presence of homologs of the Mu-like prophage CJIE1 (also known as CMLP1) is associated with increased adherence and invasion of *C. jejuni* isolates in cell culture assays [25], and DNases encoded by prophage CJIE2 and CJIE4 dramatically inhibit the natural transformation of *C. jejuni* [26, 27].

*Nitratiruptor*, a deeply-branching genus of *Campylobacterota* [6], is one of the representative culturable populations inhabiting the hydrothermal vent fields in the Okinawa Trough [3]. Previously, we characterized a novel temperate phage NrS-1 induced from *Nitratiruptor* sp. SB155-2 isolated from the Iheya North hydrothermal field of the mid-Okinawa Trough [28]. NrS-1 was taxonomically assigned to the siphovirus morphotype of *Caudoviricetes* class. The genomic analysis of the phage and its host strain suggested that the deeply-branching lineage in *Campylobacterota* experienced multiple phage infections and that the phage infection would contribute to genomic divergence of the hosts, just like the cases of pathogenic *Campylobacterota* lineages [28]. Thus, to understand the evolutional history of *Campylobacterota*, it is necessary to clarify the coevolution of temperate phages and deeply-branching *Campylobacterota* lineages in detail.

Here, we characterized four novel phages induced from a variety of *Nitratiruptor* strains isolated from the Hatoma Knoll hydrothermal field in the southern-Okinawa Trough, 450 km distant from the Iheya North hydrothermal field, and conducted comparative genomic analyses of *Nitratiruptor* strains and their temperate phages to expand our understanding of the impacts of phage infections on the genomic diversification of the deeply-branching *Campylobacterota*.

## Materials and methods

### Isolation and identification of host strain

Samples for cultivations were obtained from the Hatoma Knoll hydrothermal field located at the southern-Okinawa Trough, Japan (24°51′30″ N, 123°50′30″ E, at a depth of 1457 m) [29] using the remotely operated vehicle (ROV) *Hyper-Dolphin* during the JAMSTEC NT08-13 and NT09-11 cruises of the *R/V Natsushima* as described previously [30]. The chimney structures were divided into surface layers and interior structures, as described previously [31]. The nests of annelid polychaetes were collected from the exterior of the chimney structures. Subsamples were individually suspended in sterilized MJ synthetic seawater [32] with or without 0.05% (w/v) of neutralized sodium sulfide under an N_2_ atmosphere. The slurry of each sample was inoculated into 3 mL of MMJHS medium [31] under a gas mixture of 80% H_2_ and 20% CO_2_ (200 kPa) or 79% H_2_, 19% CO_2_, and 2% O_2_ (200 kPa) in a 15 mL glass test tube (AGC Techno Glass, Shizuoka, Japan) [11]. Then, enrichments were obtained at 37 °C and 55 °C, respectively. Isolates were obtained via three times of a dilution-to-extinction method from these enrichments. To identify the phylogenetic affiliation of the isolates, genomic DNA was extracted from each isolate using illustra bacteria genomicPrep Mini Spin Kit (Cytiva, Tokyo, Japan), and almost complete 16 S rRNA gene sequences were determined using a protocol reported previously [33].

### Prophage induction

Prophage inductions from host strains were attempted by adding various concentrations (0.001–1.0 μg mL^−1^) of mitomycin C (Nacalai Tesque, Kyoto, Japan) to the cell cultures at mid-exponential growth phase in 15 mL of a test tube. After the induction, each culture was filtered through a Millex-GP filter with a 0.22-μm pore size (Millipore, Bedford, MA, USA). The filtered sample was fixed with 0.5% glutaraldehyde. After staining with 2.5× SYBR Gold (Thermo Fisher Scientific, Waltham, MA, USA) for 15 min, the sample was filtered through a 0.02-μm Anodisc filter (Cytiva), and the prophage was trapped on the surface of the filter. Each filter was observed with an Olympus BX51 fluorescent microscope (Tokyo, Japan) at a magnification of ×1500 to check the presence or absence of viral particles [28].

### Preparation of prophage lysates

Large-scale culture (a total of approximately 1 L for each strain) was performed to prepare high-density phage stocks. Cultures were prepared in 300 mL MMJHS medium under a gas mixture of 80% H_2_ and 20% CO_2_ (200 kPa) or 79% H_2_, 19% CO_2_, and 2% O_2_ (100 kPa) with a 1 L Schott glass bottle (Schott AG, Mainz, Germany) at 55 °C. Mitomycin C was added to the cell cultures at the mid-exponential growth phase. At 18 h after the addition of mitomycin C, the cultures were filtered through a 0.22-μm pore size filter. For electron microscopy observation and genome sequencing, phage particles were concentrated using Amicon Ultra-70 and Ultra-15 centrifugal filtration units with a molecular weight cutoff of 100 kDa (Merck Millipore, Burlington, MA, USA). The concentrates were rinsed thrice with SM buffer (50 mM Tris-HCl pH 7.5, 100 mM NaCl, 10 mM MgSO_4_, 0.01% gelatin). Phage suspensions in SM buffer were further purified using CsCl gradient ultracentrifugation [34].

### Electron microscopy

An aliquot of the concentrated phage suspension was absorbed onto formvar/carbon-coated copper grids, stained with 2% uranyl acetate, and observed using a TECNAI G20 transmission electron microscope (Thermo Fisher Scientific) at 80 kV [33].

### Genome sequencing and assembly

The phage genomes were extracted from purified phage particles according to a method described previously [34]. The purified DNA was sheared using a Covaris S220 instrument (Woburn, MA, USA), following the manufacturer’s protocol to obtain a 350-bp peak. A shotgun sequencing library was constructed using the Ion Xpress Plus Fragment Library Kit (Thermo Fisher Scientific). Size selection of the library was performed using an E-gel SizeSelect 2% agarose gel (Thermo Fisher Scientific). Emulsion PCR was performed using the Ion PGM Template OT2 400 kit, and the sequencing was carried out on an Ion Torrent PGM with the Ion PGM Sequencing 400 kit and an Ion 314 Chip V2 (Thermo Fisher Scientific). The sequence reads were de novo assembled using the CLC Genomics Workbench, version 11.0 (Qiagen, Aarhus, Denmark) with default parameters. Gaps between contigs were filled by standard PCR and Sanger sequencing on a ABI 3730 capillary sequencer.

Sequencing libraries of the *Nitratiruptor* isolates were prepared with the Nextera Mate Pair Sample Preparation kit (Illumina, San Diego, CA, USA) according to the manufacturer’s instructions. Then, they were sequenced using an Illumina MiSeq version v3 reagent kit (600 cycles) with 300-bp paired-end reads on the Illumina MiSeq platform. Raw Illumina reads were sequentially processed using Trimmomatic ver. 0.39 [35] to trim the adaptor sequences and low-quality sequences. The mate-paired reads were further processed with NextClip [36] to trim the linker sequence and classify the mate-pairs. The cleaned reads were de novo assembled using the CLC Genomics Workbench, version 11.0 (Qiagen) with default parameters. Contigs were scaffolded using SSPACE [37]. The remaining sequence gaps between scaffolds were closed by PCR-based sequencing.

For methylation analysis, genomic DNA was extracted from *Nitratiruptor* sp. YY08-14 using NucleoSpin Tissue kit (Takara Bio, Kusatsu, Japan) with modifications to the manufacturer’s instruction as follows. After the cells collected by centrifugation were suspended in T1 solution provided in the kit, thiourea (final 50 μM) was added to the suspension as a free radical scavenger to prevent DNA degradation [38]. The NrS-3 phage DNA extracted from purified phage particles described above was mixed with the YY08-14 DNA, and long-read sequencing was performed on a PacBio Sequel system (Pacific Biosciences, Menlo Park, CA, USA) at DNA Link (Seoul, South Korea). The DNA fragments larger than 5 kb were prepared using a BluePippin system (Sage Sciences, Beverly, MA, USA). A PacBio SMRTbell library was constructed according to the manufacturer’s instruction. PacBio Sequel yielded a total of 1.6 million subreads (7.9 Gbp) with an N50 length of 5.13 kb. DNA base modifications were detected using the PacBio SMRT toolkit (SMRT Link v6.0.0).

### Gene annotation

Coding sequences (CDSs) in the phage genomes were identified using GeneMarkS [39] and GLIMMER [40]. The homology search was performed using the BLAST program, with a cutoff E-value of 10^–5^ against public databases (GenBank/EMBL/DDBJ). Protein functional motifs were identified using Pfam [41], InterProScan [42], and CDD [43]. Transmembrane domains and signal sequences were detected by TMHMM Server version 2.0 [44] and SignalP 5.0 Server [45]. Core genes shared by the phage genomes were determined by local ‘all against all’ BLASTP comparison for all the phage protein sequences [46]. A core gene was defined when one was harbored by all phages and had an E-value lower than 10^–5^ between any pairwise amino acid sequences.

Gene prediction and annotation of the host complete genomes were performed using the RAST server [47]. The JSpeciesWS was used to calculate average nucleotide identity based on BLAST + (ANIb) [48]. The host genome sequences were also analyzed to identify the presence of prophages with PHASTER [49]. Sequence similarity search against dataset from the Restriction Enzyme Database (REBASE) [50] was performed using the BLAST program with a cutoff E-value of 10^–5^. Clustered regularly interspaced short palindromic repeats (CRISPRs) and their associated proteins (Cas) were identified using CRISPRCasFinder [51].

### Phylogenetic analysis

The phylogenetic tree of 16S rRNA gene was constructed using the maximum-likelihood (ML) method with 100 resampling bootstrap analyses in MEGA11 software [52]. Phylogenetic trees of the phages were constructed using the Genome-BLAST Distance Phylogeny method (GBDP) implemented in VICTOR [53] under settings (distance formula *d6*) recommended for prokaryotic viruses.

## Results and discussion

### Isolation of *Nitratiruptor* strains and their genomes

**Fig. 1:**
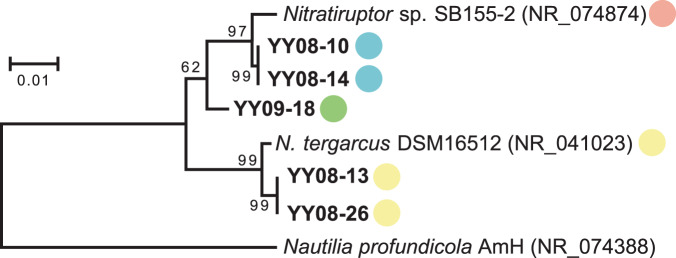
A maximum-likelihood phylogenetic tree based on almost complete 16 S rRNA gene sequences of representative members of the genus *Nitratiruptor* using 1408 to 1420 nucleotides. Bootstrap analysis was performed with 100 repetitions. Boldface type indicates *Nitratiruptor* strains obtained in this study. Each strain was color-coded based on the species-level-definition (95%) of ANI identity. GenBank accession numbers of 16 S rRNA gene sequences are given in parentheses. *Nautilia profundicola* AmH was used as an outgroup. The scale bar indicates a 0.01 change per nucleotide.

The present study isolated five new *Nitratiruptor* strains from four different samples collected in the Hatoma Knoll hydrothermal field located at the southern-Okinawa Trough (Table 1 and Fig. 1). *Nitratiruptor* strain SB155-2 with the temperate phage NrS-1 and *N. tergarcus* DSM16512 with incomplete prophage regions (scores of PHASTER < 40) were also used as reference strains. They were isolated from the Iheya North hydrothermal field of the mid-Okinawa Trough [11, 54]. The isolated strains grew with H_2_ as an electron donor and NO_3_^−^ or O_2_ as an electron acceptor at 37 °C or 55 °C. The complete genome sequences were obtained for all five *Nitratiruptor* strains (Table 1 and Supplementary Table S1). The genomes were 1.73 to 1.89 Mbp in length and predicted to harbor 1808 to 2007 CDSs. Genomic G + C content ranged from 37.1% to 39.1%. All the isolates in this study had one circular plasmid of 24–35 kbp in length, while the plasmid was absent in strain SB155-2 [11]. Genes related to prophages and restriction-modification systems were not detected on their plasmids. The average nucleotide identity (ANI) of genome sequences among *Nitratiruptor* strains ranged from 73.29% to 100% (Supplementary Table S2). Based on the species-level-definition (95%) of ANI identity [55], the *Nitratiruptor* strains tentatively fell into four species: (1) SB155-2; (2) YY08-10 and YY08-14; (3) YY09-18; and (4) *N. tergarcus* (DSM16512, YY08-13, and YY08-26). The 16S rRNA gene sequences of strains YY08-10, YY08-14, and YY09-18 shared more than 97% similarity with SB155-2; however, they shared low ANIb values of 73–87% with each other, except for that between strains YY08-10 and YY08-14. Strains YY08-10 and YY08-14 have almost the same genome sequence except for an inversion of approximately 50 kbp region. The *N. tergarcus* group (strains DSM16512, YY08-13, and YY08-26) showed high ANIb values of more than 95%. Strains YY08-13 and YY08-26 shared almost identical sequences except for several nucleotide substitutions and insertions/deletions (indels).

**Table 1:** General isolation, physiological properties and genomic features of *Nitratiruptor* strains and general characteristics of their inducible phages.

Characteristics	*Nitratiruptor* strain						
	SB155-2	DSM16512^a^	YY08-10	YY08-14	YY09-18	YY08-13	YY08-26
Isolation site							
Hydrothermal vent site	Iheya NBC	Iheya NBC	Hatoma 189-1	Hatoma 189-1	Hatoma Oritori	Hatoma C-2	Hatoma C-2
Sample description	ISCS^b^	Chimney inside	Chimney surface	Chimney surface	Polychaete nest	Chimney inside	Chimney surface
Cultivation							
Headspace gas^c^	H_2_/CO_2_ (+O_2_)	H_2_/CO_2_ (+O_2_)	H_2_/CO_2_/O_2_	H_2_/CO_2_	H_2_/CO_2_	H_2_/CO_2_	H_2_/CO_2_
Temp. (°C)	55	55	55	55	37	55	55
Genome							
Chromosome							
Size (bp)	1,877,931	1,894,691	1,792,750	1,806,716	1,732,647	1,893,367	1,893,369
G + C content (%)	39.7	36.9	39.1	39.0	39.1	37.1	37.1
No. of protein coding sequences	1918	1906	1872	1881	1808	2005	2007
No. of rRNA operons	3	1	3	3	3	3	3
No. of tRNAs	45	39	45	45	42	43	43
Plasmid							
Size (bp)	–	34,742	27,067	27,067	24,473	35,300	35,300
G + C content (%)	–	34.3	31.9	31.9	35.8	36.0	36.0
No. of protein coding sequences	–	35	31	31	27	35	35
CRISPR	0	2	0	0	2	1	1
Inducible temperate phage							
Phage name	NrS-1	–	NrS-2	NrS-3	–	NrS-4	NrS-5
Induction							
Mitomycin C conc. (μg mL^−1^)	0.01	–	0.001	0.01	–	0.1	0.01
Morphology							
Capsid dia. (nm)	64	–	63	55	–	61	61
Tail (nm)	210 × 10	–	213 × 10	210 × 10	–	337 × 10	337 × 10
Morphotype	siphovirus	–	siphovirus	siphovirus	–	siphovirus	siphovirus
Genome							
Size (bp)	37,159	–	40,465	40,036	–	43,030	43,030
G + C content (%)	39.7	–	39.2	39.2	–	39.0	39.0
GenBank accession no.							
Chromosome	AP009178.1	FWWZ01000001.1	AP023057.1	AP023061.1	AP023065	AP023059.1	AP023063.1
Plasmid	–	FWWZ01000002.1	AP023058.1	AP023062.1	AP023066	AP023060.1	AP023064.1
Temperate phage	AB746912.1	–	LC545443.1	LC545444.1	–	LC545445.1	LC545446.1
References	[11, 28]	[54]	This study	This study	This study	This study	This study

^a^The genome of DSM16512 deposited as an almost completed scaffold genome (GCF_900176045.1) was used as a reference.

^b^ISCS indicates ‘in situ colonization systems’ deployed on the actively venting sulfide mound [31].

^c^H_2_/CO_2,_ H_2_:CO_2_ = 80:20; H_2_/CO_2_/O_2_, H_2_:CO_2_:O_2_ = 79:19:2; H_2_/CO_2_ (+O_2_), H_2_:CO_2_ = 80:20 or H_2_:CO_2_:O_2_ = 79:19:2.

### Induction of *Nitratiruptor* temperate phages

Phage induction was tested for all five isolates by adding various concentrations of mitomycin C (0.001–1.0 μg mL^−1^). Based on the microscopic observation, virus-like particles were found in the culture of four strains (YY08-10, YY08-14, YY08-13, and YY08-26) (Table 1 and Supplementary Fig. S1). The optimal concentration of mitomycin C for the phage induction of each host strain varied from 0.001 to 0.1 μg mL^−1^ (Table 1). As in the case of SB155-2, the spontaneous induction of virus-like particles was also observed during the growth of these host strains (Supplementary Fig. S1). No virus-like particle was found in the YY09-18 culture. This result is consistent with the absence of any complete prophage region in the genome of YY09-18, where three incomplete prophage regions were identified (scores of PHASTER < 70).

### Morphology of *Nitratiruptor* phages

**Fig. 2:**
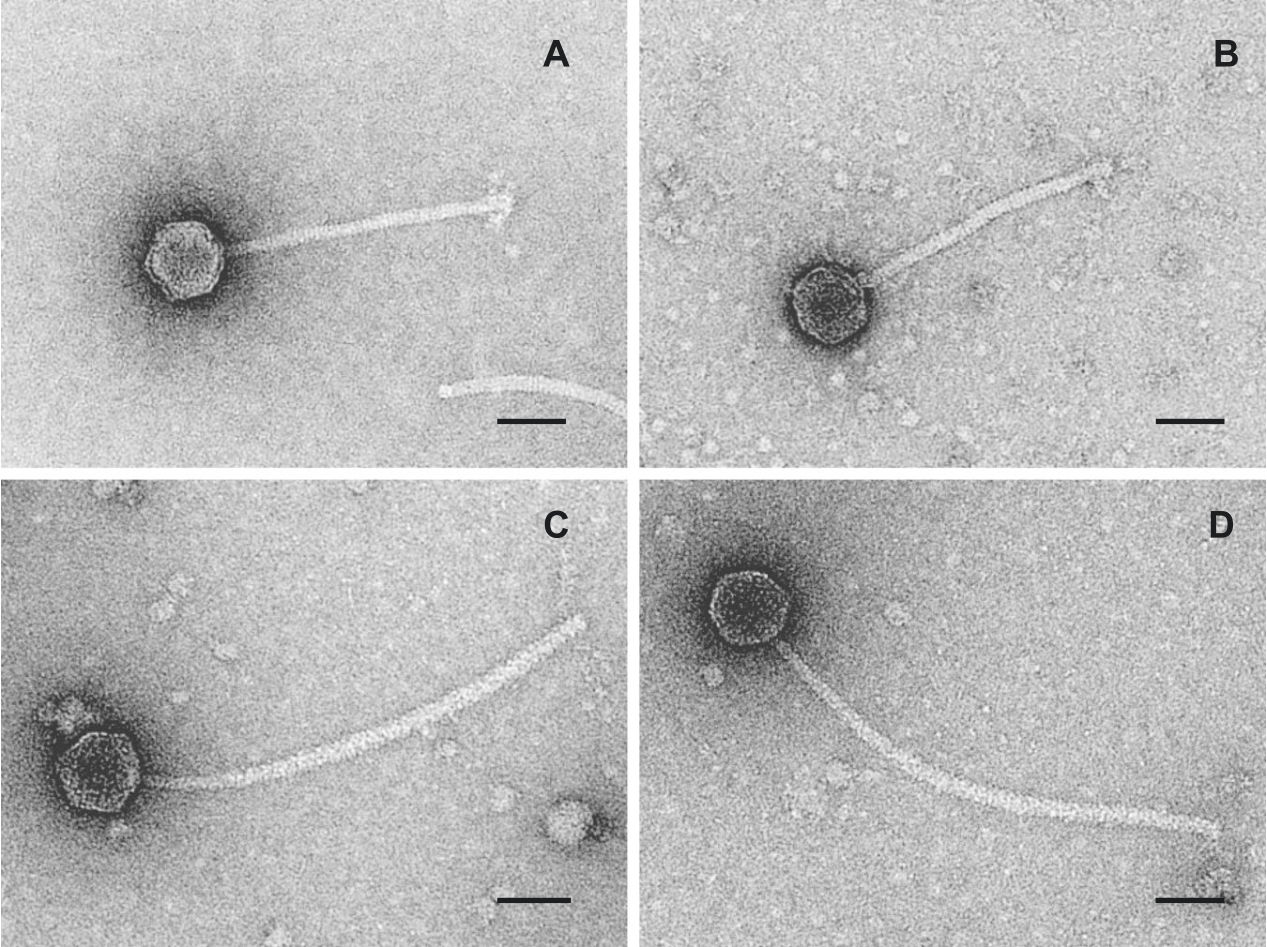
Transmission electron micrographs of temperate phages isolated in this study. The bar indicates 50 nm. **A** NrS-2 from *Nitratiruptor* sp. YY08-10, (**B**) NrS-3 from *Nitratiruptor* sp. YY08-14, (**C**) NrS-4 from *Nitratiruptor* sp. YY08-13, and (**D**) NrS-5 from *Nitratiruptor* sp. YY08-26.

The phage particles induced from *Nitratiruptor* sp. strain YY08-10 had an isometric head of approximately 63 nm and a flexible non-contractile tail of 213 nm × 10 nm (*n* = 30) (Fig. 2A and Table 1). Those from strain YY08-14 consisted of an isometric head of approximately 55 nm and a flexible non-contractile tail of 210 nm × 10 nm (Fig. 2B and Table 1). Both phage particles from strains YY08-10 and YY08-14 were similar to the NrS-1 phage induced from *Nitratiruptor* sp. SB155-2 [28]. The phage particles from strains YY08-13 and YY08-26 showed a very similar morphology with an isometric head (approximately 61 nm) and a very long, flexible non-contractile tail (~337 nm × 10 nm) (Fig. 2C, D and Table 1). All these four phages had a typical siphovius morphology. The phages were named NrS-2 (from YY08-10), NrS-3 (from YY08-14), NrS-4 (from YY08-13), and NrS-5 (from YY08-26), respectively.

### Genomic features of *Nitratiruptor* phages

#### NrS-2 and NrS-3

**Fig. 3:**
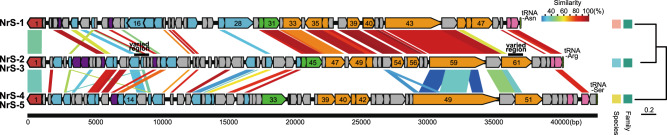
Genome organization and whole-genome comparison of *Nitratiruptor* temperate phages (left) and their genome-based phylogenetic tree (right). Arrows represent predicted genes and coding directions. Colors show different functional groups of gene products: red, integration; light blue, genetic switch, replication and recombination; green, packaging; orange, structural protein; pink, cell lysis; purple, other function; and gray, unknown. Gene homology between temperate phages was visualized by the degree of sequence identity between a pair of NrS-2 and NrS-3 shared 100% identical sequences except for two varied regions (black bar). The genomes of NrS-4 and NrS-5 were 100% identical to each other. The phylogenetic tree was constructed by VICTOR using the complete genome sequences of these temperate phages. Colors indicate the classification at the species and family level.

The circularly assembled double-stranded DNA genomes of NrS-2 and NrS-3 phages consist of 40,465 bp and 40,036 bp with average coverage of 102 and 68, respectively (Table 1, Fig. 3 and Supplementary Table S1). Their complete genomes are found in their host genomes (strain YY08-10, position 282,568-323,032 and strain YY08-14, position 282,568-322,603). Both phages were integrated into the tRNA^Arg^ gene in their host genomes, whereas the previously identified phage NrS-1 was in the tRNA^Asn^ gene [28]. Their genomes showed an identical sequence except for two regions, and the G + C contents were 39.2%, which are similar to those of their hosts (39.1% and 39.0%). A total of 66 putative CDSs were identified in each genome (Fig. 3 and Supplementary Tables S3, S4). One of the distinct regions is an ~900 bp region encoding NRS2_17 (NRS3_17) to NRS2_19 (NRS3_19). NRS2_17 and NRS3_17 have a common sequence at the C-terminus, while those had distinct domains at the N-terminus: Phage_pRha (PF09669, NrS-2) and a P22_AR_N (PF10547, NrS-3). NRS2_18 and NRS2_19 of NrS-2 had 63% and 94% amino acid sequence identities with NRS3_18 and NRS3_19 of NrS-3, respectively. The other one was a part of NRS2_61 and NRS3_61 (tail fiber protein). In a comparison of NRS2_61 and NRS3_61, a region of 151 amino acids, equivalent to three out of six NHL repeats in the NHL domain, was absent in NRS3_61.

#### NrS-4 and NrS-5

The genomes of NrS-4 and NrS-5 were comprised of 43,030 bp with a genomic G + C content of 39.0% (Table 1 and Fig. 3). The assembled genomes have average coverages of 157 and 113, respectively (Supplementary Table S1). Although there were differences in isolation sources of their host strains and optimum mitomycin C concentrations for their induction (Table 1), the genome sequences of NrS-4 and NrS-5 were 100% identical to each other (Fig. 3), and the ANIb between their host strains was 100.00 (Supplementary Table S2). Therefore, we chose NrS-4 and *Nitratiruptor* strain YY08-13 as the representative phage and host, respectively, for further analyses. The NrS-4 genome was found in position 497,501-540,530 of the host YY08-13 genome and integrated into the tRNA^Ser^ gene in the host genome. A total of 59 CDSs were predicted from the NrS-4 genome (Fig. 3 and Supplementary Table S5). Based on the BLASTP search results, most of the CDSs (76%) shared significant similarities (*E*-value of <10^−5^) with sequences in the public databases. The length of tail tape measure protein (NRS4_49; 2,178 amino acids) in NrS-4 was longer than its homolog in NrS-1 (NRS1_43; 1,421 amino acids) [28], NrS-2 and NrS-3 (NRS2_59 and NRS3_59; 1,421 amino acids). The difference in gene length was in good agreement with the electron microscopy observation that the tail length of phage NrS-4 is about one and a half times as long as that of NrS-1, NrS-2, and NrS-3 (Fig. 2) [56].

### Comparison of phage genome structure

NrS-2 and NrS-3 shared 33 CDSs with NrS-1 (*E*-value of <10^−5^), which accounted for 50% of their CDSs (Fig. 3). The amino acid identities between the proteins encoded by NRS1_30 to NRS1_44 in NrS-1 and NRS2_44 to NRS2_60 in NrS-2 (NRS3_44 to NRS3_60 in NrS-3) in the rightward regions, including the genes of the phage terminases, structural proteins, and lysis proteins, ranged from 70 to100% (average 87%), while the ‘functional genes’ in the leftward direction shared more variable identities between these phages. Phage genome comparison showed no continuous co-linear genomic structure between NrS-4 and NrS-1 at the nucleotide levels, whereas the NrS-4 genome sequence showed weak co-linearity with the NrS-2 genome in the leftward regions (Fig. 3).

To determine the conserved core genes of the *Nitratiruptor* phages, we performed a local BLASTP comparison in any CDSs among them. They shared 12 potential core genes, including integrase, Cro, two structural proteins (tape measure protein and tail fiber protein), lysozyme, MazG, and six genes with unknown functions (Supplementary Table S6). The result suggests that these core genes are essential for the propagation of phages in the *Nitratiruptor* population, as is the case of other temperate siphoviruses infecting thermophilic *Marinitoga* bacteria from deep-sea hydrothermal vents [57]. The abundance of unique proteins in NrS-4 (58%) was higher than that in the other two phages (31% in NrS-1 and 35% in NrS-2). Most of unique proteins in NrS-4 were functionally unknown proteins and structural proteins located in the rightward regions of the genome (Supplementary Table S5).

A phylogenetic analysis of these *Nitratiruptor* phages using the VICTOR web service showed that they were discriminated as three different species, although all the phages clustered to the same family (Fig. 3). The classification of phage species was consistent with the classification of the host. Considering the high host specificity of phages [58], this result may represent that *Nitratiruptor* phages diversified by acquiring unique genes while sharing many potential core genes along the species divergence of *Nitratiruptor*.

### Phage-host interactions: distribution of phage homologous genes in *Nitratiruptor* genomes

The representative four species of *Nitratiruptor* had very similar genomes with a highly conserved organization (Supplementary Fig. S2) as in the cases of other *Campylobacterota* genera such as *Lebetimonas* [59] and *Campylobacter* [20]. A major difference in genome organization among the *Nitratiruptor* strains was the distribution pattern of genomic islands. A total of five genomic islands were identified in the genome of SB155-2, and one of those (GI1) was the prophage sequence of NrS-1 identified previously [28]. Mobile genetic elements, such as genomic islands or prophages, are powerful agents that affect the diversity and evolution of microbial communities by horizontal gene transfer [60]. The *Nitratiruptor*-associated prophages also may be one of the major elements that promote genomic diversity in *Nitratiruptor*, as in the case of *Campylobacter* strains [21–24].

All the CDSs in the four *Nitratiruptor* phage genomes were subjected to a BLAST search against the *Nitratiruptor* genomes, including that of strain YY09-18 without the production of viral particles, in order to understand the distribution patterns of CDSs encoding homologs of phage proteins in the *Nitratiruptor* genomes. Some homologs, including integrases (phage core gene), phage transcriptional regulators, and ssDNA binding protein, were found throughout the non-prophage regions of the *Nitratiruptor* genomes (Supplementary Table S7). The homologs of transcriptional regulator (NRS4_25) of NrS-4 were detected in the non-prophage regions of not only the YY08-13 genome of its host but SB155-2 genome, which was isolated from another vent and classified as a different species, and exhibited more than 80% sequence identity. Furthermore, the homologs of ssDNA binding proteins (NRS2_24 and NRS4_13) were found in all *Nitratiruptor* genomes. This result suggested that *Nitratiruptor* was infected by multiple lineages of temperate viruses. Considering the conservative genome organization among the genus *Nitratiruptor* and the random distribution of the phage gene homologs, the acquisition of phage genes likely plays an important role in the diversification of their genomes. Further studies are required to understand the impacts of phage genes on the host evolution and niche adaptation as in the cases of defective phages [61].

### Phage-host interactions: restriction-modification and CRISPR

“Arms race” between bacteria and their phages rapidly prompts both the evolution of bacterial defense systems against phage attacks and the further counter systems of phages [58, 62, 63]. Restriction-modification systems are one of the principal host defense mechanisms against phage infections [58, 62]. However, several phages can protect their own genomes from the host restriction cleavage through methylation [58]. Among the five *Nitratiruptor* phages, only NrS-2 and NrS-3 have a gene for methyltransferase (NRS2_07, NRS3_07), showing high similarity to that of *Bacteroidales* bacterium within *Bacteroidota* (Supplementary Tables S3, S4). Sequence similarity searches against known non-putative methyltransferase genes stored in REBASE showed that these genes presented the highest sequence similarity to the Type II N6-adenine DNA methyltransferase (M.Dor12838I) recognizing R(=A/G)GATCY(=T/C) from *Desulfomicrobium orale* DSM 12838. In addition, they have a gene for a homolog of type II restriction endonuclease BglII (NRS2_08, NRS3_08) adjacent to their methyltransferase (Supplementary Tables S3, S4). The BglII homolog was absent not only in their host genomes, but in the genomes of all the previously known strains of *Campylobacterota* and their phages. Thus, the restriction enzymes encoded by NrS-2 and NrS-3 were likely acquired via horizontal gene transfer events from other groups of bacteria. Based on the PacBio data of the genomes of *Nitratiruptor* sp. YY08-14 strain and its phage NrS-3, four methylated motifs were identified (Supplementary Table S8), of which the RGATCY motif that covers the BglII recognition sequence (AGATCT) was 100% methylated in their genomes. One of the four methyltransferases encoded by *Nitratiruptor* sp. YY08-14 was predicted to recognize the GCNGC motif identified using a PacBio sequence, while the other three were undetermined. Although the function of the restriction-modification system of NrS-2 and NrS-3 in vivo has not yet been proven, this phage restriction enzyme, in addition to the immunity repressor CI, likely functions as a unique defense mechanism against other infecting phages and foreign DNA and provides an advantage for their hosts. A similar function of a putative type II restriction enzyme in *Burkholderia* prophage has also been predicted [64].

CRISPR-Cas systems are also a widespread phage resistance mechanism in prokaryotes [58]. CRISPR spacer sequences between the direct repeats are derived from the genomes of phages that infected the host cell in the past [58]. To explore the past infections of the *Nitratiruptor* phages, the six *Nitratiruptor* genomes were analyzed using CRISPRCasFinder [51].

**Fig. 4:**
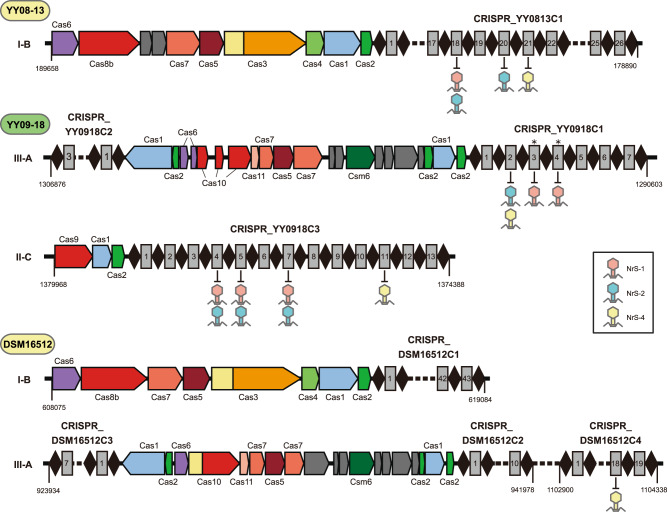
CRISPR-Cas systems identified in the genomes of *Nitratiruptor* strains and CRISPR spacers targeting their temperate phages. Cas genes are colored according to Makarova et al. [73]. The direct repeats and spacers are indicated as black diamonds and numbered as gray rectangles, respectively.

CRISPR-Cas systems were identified in the genomes of strains YY08-13, YY09-18, and DSM16512, whereas no CRISPR-Cas systems were found in the genomes of strains SB155-2, YY08-10, and YY08-14. Strain YY08-13 has a subtype I-B CRISPR-Cas system with an array containing 26 spacers (CRISPR_YY0813C1) (Fig. 4 and Supplementary Table S9). Strain YY09-18 harbored subtypes III-A and II-C CRISPR-Cas systems with three array loci and a total of 23 spacers (CRISPR_YY0918C1-C3). Strain DSM16512 harbored subtypes I-B and III-A CRISPR-Cas systems with four arrays and a total of 79 spacers (CRISPR_DSM16512C1-C4). The CRISPR spacer sequences of those three strains are not shared with each other. The genes in the Cas operons of the YY08-13 genome shared significant similarities with the homologs of *Deferribacter desulfuricans* SSM1 in *Deferribacterota* and the deep-sea vent and terrestrial *Aquificota* members. On the other hand, the genes in both Cas operons of the YY09-18 genome shared significant similarities with the deep-sea vent and pathogenic *Campylobacterota* strains. Considering the high horizontal mobility of CRISPR-Cas systems [65], these CRISPR-Cas systems may be acquired and retained via horizontal gene transfer from other lineages individually.

A BLAST search showed that three CRISPR spacers in strain YY08-13, seven CRISPR spacers in strain YY09-18, and one CRISPR spacer in strain DSM16512 matched genome sequences of *Nitratiruptor* phages analyzed in this study (Fig. 4 and Table 2). The spacer YY0918C1_s2 of strain YY09-18 without the prophage was completely identical to a part of CDS coding a DNA-binding protein (NRS2_13). These observations indicate that *Nitratiruptor* CRISPR-Cas systems are functional, and these strains have undergone multiple infections of the *Nitratiruptor* phages or their relatives. The protospacers were all located in the CDS sequences in the phage genomes, except for DSM16512C4_s18 of DSM16512. Especially, all three spacers in strain YY08-13 and one spacer in strain YY09-18 (YY0918C2_s7) were similar to the sequences in genes for tape measure proteins (core gene) of the three phages. These functional commonalities between the spacers strongly suggest that these genes are essential for the phage propagation and the defense of *Nitratiruptor* strains against the phage infection.

**Table 2:** Sequence homology between spacers and protospacers in phage genomes.

*Nitratiruptor* strain	CRISPR array	Spacer name	Target phage		
			NrS-1 (host SB155-2)	NrS-2 (host YY08-10)	NrS-4 (host YY08-13)
YY08-13 (YY08-26)	CRISPR_YY0813C1	YY0813C1_s18	CATCAAATCCATCACAAA**G**GCGATCGGTGATGA**A**TT (34/36) NRS1_43* (Tape measure protein)	CATCAAATCCATCACAAAAGC**A**ATCGGTGATGAGTT (35/36) NRS2_59* (Tape measure protein)	
		YY0813C1_s20		C**T**TCTTTTGTTGTCTTTTTGGT**G**G**TG**TTTTTCACATT (33/37) NRS2_59* (Tape measure protein)	
		YY0813C1_s21			TGCTGAAAAA**C**TTTCAAAAGCAAATATCACAACACA (35/36) NRS4_49* (Tape measure protein)
YY09-18	CRISPR_YY0918C1	YY0918C1_s2		CGCAATCTTTGCGCAGTACGCAATGATCATAT (32/32) NRS2_13* (DNA-binding protein)	CGCA**T**TCTTTGCGCAGTACGCAATG**G**TC**G**TAT (29/32) NRS4_09* (DNA-binding protein)
		YY0918C1_s3	TCATCGAC**C**TTAAGGTGTTTTTC**G**CTGTACGGGTTGAG**T**T (37/40) NRS1_27 (CII)		
		YY0918C1_s4	TCATCGAC**C**TTAAGGTGTTTTTC**G**CTGTACGGGTTGAG**T**T (37/40) NRS1_27 (CII)		
	CRISPR_YY0918C3	YY0918C3_s4	T**C**CT**G**TT**G**GCATAAAA**C**CTCGCTTTCTCCT (26/30) NRS1_33 (Phage head morphogenesis protein)	TTCTATTTGCATAAAA**C**CTCGCTTT**T**TC**T**T (27/30) NRS2_47 (Phage head morphogenesis protein)	
		YY0918C3_s5	GAACTTTACAAACTC**T**ACTTCATCATCTTC (29/30) NRS1_33 (Phage head morphogenesis protein)	**A**AACTTTACAAACTCCACTTCATCATCTTC (29/30) NRS2_47 (Phage head morphogenesis protein)	
		YY0918C3_s7	TAAAACTTCGAGC**G**TA**C**GGACTTGAACCTA (28/30) NRS1_43* (Tape measure protein)	TAAAGCTTCGAGCATATGGACTCGAACCTA (28/30) NRS2_59* (Tape measure protein)	
		YY0918C3_s11			**G**CATAGG**T**ATATTGGT**G**TTGGGGCTTGCGTA (28/31) NRS4_55 (Hypothetical protein)
DSM16512	CRISPR_DSM16512C4	DSM16512C4_s18			CTGCCAAAGGGG**C**TGAGTTGTGATAGGACTCAG (32/33) non-coding region between NRS4_19 and NRS4_20

Asterisks (*) indicates core genes shared by *Nitratiruptor* phages.

Boldface type and underline indicate nucleotide substitutions between the spacer sequences and the phage protospacers.

In contrast to strain YY09-18 lacking prophages, strain YY08-13 has a spacer sequence (YY0813C1_s21) almost identical to a protospacer in its own prophage NrS-4 (Table 2). The type I-B CRISPR-Cas system is known to contain many self-targeting spacers against prophages; however, genetic degradation of CRISPR-Cas systems appears to be rare, suggesting that alternative mechanisms such as anti-CRISPR proteins and CRISPR target mutations allow to escape from lethal effects of auto-immunity [66]. We found no anti-CRISPR proteins in the genomes of strain YY08-13 (and NrS-4) using ArcFinder [67]. In the type I-B CRISPR-Cas system, a conserved 5′ protospacer-adjacent motif (5′ PAM) and a SEED sequence were found to be essential for the recognition of the protospacer [68, 69]. The 5′ PAM sequences immediately upstream of protospacers in *Nitratiruptor* phage genomes exhibited the conserved 5′-TW(=T/A)A-3′ motif. We found one nucleotide mismatch at position 11 in the protospacer of NrS-4. Although the SEED region of the type I-B CRISPR-Cas system of *Nitratiruptor* is unknown, the NrS-4 phage can propagate in the cells of strain YY08-13 by switching from the lysogenic to the lytic cycle (Supplementary Fig. S1) as observed in NrS-1 phage [28]. Thus, NrS-4 might be able to escape from the host’s CRISPR recognition due to one base pair mutation of the corresponding gene in the phage genome, representing one of the major driving forces for the evolution of bacteriophage mutants [70–72]. This is the first report of annotated CRISPR-associated features in bacteria and their temperate phages isolated from deep-sea hydrothermal environments, and it was suggested that *Nitratiruptor* has undergone multiple phage infections and co-evolved in a repeated arms race with their phages.

## Conclusions

We identified and characterized four novel temperate siphoviruses (NrS-2, NrS-3, NrS-4, and NrS-5) that infected chemolithoautotrophic deep-sea *Campylobacterota*, *Nitratiruptor* strains. The comparative genomic analysis for these phages and a previously reported temperate phage (NrS-1) revealed that these phages have diversified by acquiring unique genes while inheriting many common genes from their ancestral *Nitratiruptor* phage, suggesting that phages co-evolved along the species divergence of *Nitratiruptor*. *Nitratiruptor* was infected multiple times by diverse phages and diversified by the acquisition of prophage genes and various immune mechanisms (phage repressor, restriction-modification system, and CRISPR-Cas systems). In addition, the coevolutionary ‘arms race’ between phages and their hosts may have driven the genomic diversification and ecophysiological adaptation of both phages and their hosts in the highly diverse and dynamic habitats of deep-sea hydrothermal environments. Further investigation of the host specificity of phages and the expansion of host-phage libraries will undoubtedly lead to a better understanding of the role of temperate phages in the species divergence and speciation of *Nitratiruptor*.

## Supplementary Information

Table S1

Table S2

Table S3

Table S4

Table S5

Table S6

Table S7

Table S8

Table S9

Supplemental Figures

## Data Availability

The complete nucleotide sequences are available under accession numbers AP023057 to AP023066 for *Nitratiruptor* strains and LC545443 to LC545446 for temperate phages (Table 1), and BioProject number PRJDB9639. The BioSample numbers and DRA accession numbers for the raw data are listed in Supplementary Table S1.

## References

[1] Jeanthon C (2000). Molecular ecology of hydrothermal vent microbial communities. Antonie Van Leeuwenhoek.

[2] Nercessian O, Reysenbach A-L, Prieur D, Jeanthon C (2003). Archaeal diversity associated with in situ samplers deployed on hydrothermal vents on the East Pacific Rise (13^o^N). Environ Microbiol.

[3] Nakagawa S, Takai K, Inagaki F, Hirayama H, Nunoura T, Horikoshi K (2005). Distribution, phylogenetic diversity and physiological characteristics of epsilon-*Proteobacteria* in a deep-sea hydrothermal field. Environ Microbiol.

[4] Brazelton WJ, Schrenk MO, Kelley DS, Baross JA (2006). Methane- and sulfur-metabolizing microbial communities dominate the Lost City hydrothermal field ecosystem. Appl Environ Microbiol.

[5] Takai K, Nakagawa S, Reysenbach A-L, Hoek J. Microbial ecology of mid-ocean ridges and back-arc basins. In: Christie DM, Fisher CR, Lee S-M, Givens S, editors. Geophysical Monograph Series. 2006. Washington, D. C.: American Geophysical Union; 2006. pp. 185–213.

[6] Nakagawa S, Takai K (2008). Deep-sea vent chemoautotrophs: diversity, biochemistry and ecological significance. FEMS Microbiol Ecol.

[7] Jørgensen BB, Boetius A (2007). Feast and famine—microbial life in the deep-sea bed. Nat Rev Microbiol.

[8] Campbell BJ, Engel AS, Porter ML, Takai K (2006). The versatile ε-proteobacteria: key players in sulphidic habitats. Nat Rev Microbiol.

[9] Oren A, Garrity GM (2021). Valid publication of the names of forty-two phyla of prokaryotes. Int J Syst Evol Microbiol.

[10] Nakagawa S, Takaki Y. Nonpathogenic Epsilonproteobacteria. Encyclopedia of Life Sciences (eLS). Chichester, UK: John Wiley & Sons, Ltd; 2009.

[11] Nakagawa S, Takaki Y, Shimamura S, Reysenbach A-L, Takai K, Horikoshi K (2007). Deep-sea vent *ε*-proteobacterial genomes provide insights into emergence of pathogens. Proc Natl Acad Sci USA.

[12] Porcelli I, Reuter M, Pearson BM, Wilhelm T, van Vliet AH (2013). Parallel evolution of genome structure and transcriptional landscape in the Epsilonproteobacteria. BMC Genom.

[13] Zhang Y, Sievert SM (2014). Pan-genome analyses identify lineage- and niche-specific markers of evolution and adaptation in *Epsilonproteobacteria*. Front Microbiol.

[14] Vorwerk H, Huber C, Mohr J, Bunk B, Bhuju S, Wensel O (2015). A transferable plasticity region in *Campylobacter coli* allows isolates of an otherwise non-glycolytic food-borne pathogen to catabolize glucose. Mol Microbiol.

[15] Jiang SC, Kellogg CA, Paul JH (1998). Characterization of marine temperate phage-host systems isolated from Mamala Bay, Oahu, Hawaii. Appl Environ Microbiol.

[16] Paul JH (2008). Prophages in marine bacteria: dangerous molecular time bombs or the key to survival in the seas?. ISME J.

[17] Harrison E, Brockhurst MA (2017). Ecological and evolutionary benefits of temperate phage: what does or doesn’t kill you makes you stronger. BioEssays.

[18] Fouts DE, Mongodin EF, Mandrell RE, Miller WG, Rasko DA, Ravel J (2005). Major structural differences and novel potential virulence mechanisms from the genomes of multiple *Campylobacter* species. PLoS Biol.

[19] Zhang M, He L, Li Q, Sun H, Gu Y, You Y (2010). Genomic characterization of the Guillain-Barre syndrome-associated *Campylobacter jejuni* ICDCCJ07001 isolate. PLoS ONE.

[20] Miller WG, Yee E, Chapman MH, Smith TPL, Bono JL, Huynh S (2014). Comparative genomics of the *Campylobacter lari* group. Genome Biol Evol.

[21] Parker CT, Quiñones B, Miller WG, Horn ST, Mandrell RE (2006). Comparative genomic analysis of *Campylobacter jejuni* strains reveals diversity due to genomic elements similar to those present in *C. jejuni* strain RM1221. J Clin Microbiol.

[22] Clark CG, Ng L-K (2008). Sequence variability of *Campylobacter* temperate bacteriophages. BMC Microbiol.

[23] Quiñones B, Guilhabert MR, Miller WG, Mandrell RE, Lastovica AJ, Parker CT (2008). Comparative genomic analysis of clinical strains of *Campylobacter jejuni* from South Africa. PLoS ONE.

[24] Clark CG, Chen C, Berry C, Walker M, McCorrister SJ, Chong PM (2018). Comparison of genomes and proteomes of four whole genome-sequenced *Campylobacter jejuni* from different phylogenetic backgrounds. PLoS ONE.

[25] Clark CG, Grant CC, Pollari F, Marshall B, Moses J, Tracz DM (2012). Effects of the *Campylobacter jejuni* CJIE1 prophage homologs on adherence and invasion in culture, patient symptoms, and source of infection. BMC Microbiol.

[26] Gaasbeek EJ, Wagenaar JA, Guilhabert MR, Wösten MMSM, van Putten JPM, van der Graaf-van Bloois L (2009). A DNase encoded by integrated element CJIE1 inhibits natural transformation of *Campylobacter jejuni*. J Bacteriol.

[27] Gaasbeek EJ, Wagenaar JA, Guilhabert MR, van Putten JPM, Parker CT, van der Wal FJ (2010). Nucleases encoded by the integrated elements CJIE2 and CJIE4 inhibit natural transformation of *Campylobacter jejuni*. J Bacteriol.

[28] Yoshida-Takashima Y, Takaki Y, Shimamura S, Nunoura T, Takai K (2013). Genome sequence of a novel deep-sea vent epsilonproteobacterial phage provides new insight into the co-evolution of *Epsilonproteobacteria* and their phages. Extremophiles.

[29] Glasby GP, Notsu K (2003). Submarine hydrothermal mineralization in the Okinawa Trough, SW of Japan: an overview. Ore Geol Rev.

[30] Yoshida-Takashima Y, Nunoura T, Kazama H, Noguchi T, Inoue K, Akashi H (2012). Spatial distribution of viruses associated with planktonic and attached microbial communities in hydrothermal environments. Appl Environ Microbiol.

[31] Takai K, Inagaki F, Nakagawa S, Hirayama H, Nunoura T, Sako Y (2003). Isolation and phylogenetic diversity of members of previously uncultivated ε-Proteobacteria in deep-sea hydrothermal fields. FEMS Microbiol Lett.

[32] Sako Y, Takai K, Ishida Y, Uchida A, Katayama Y (1996). *Rhodothemus obamensis* sp. nov., a modern lineage of extremely thermophilic marine bacteria. Int J Syst Bacteriol.

[33] Yoshida M, Yoshida-Takashima Y, Nunoura T, Takai K (2015). Genomic characterization of a temperate phage of the psychrotolerant deep-sea bacterium *Aurantimonas* sp. Extremophiles.

[34] Yoshida T, Takashima Y, Tomaru Y, Shirai Y, Takao Y, Hiroishi S (2006). Isolation and characterization of a cyanophage infecting the toxic cyanobacterium *Microcystis aeruginosa*. Appl Environ Microbiol.

[35] Bolger AM, Lohse M, Usadel B (2014). Trimmomatic: a flexible trimmer for Illumina sequence data. Bioinformatics.

[36] Leggett RM, Clavijo BJ, Clissold L, Clark MD, Caccamo M (2014). NextClip: an analysis and read preparation tool for Nextera long mate pair libraries. Bioinformatics.

[37] Boetzer M, Henkel CV, Jansen HJ, Butler D, Pirovano W (2011). Scaffolding pre-assembled contigs using SSPACE. Bioinformatics.

[38] Corkill JE, Graham R, Hart CA, Stubbs S (2000). Pulsed-field gel electrophoresis of degradation-sensitive DNAs from *Clostridium difficile* PCR ribotype 1 strains. J Clin Microbiol.

[39] Besemer J (2001). GeneMarkS: a self-training method for prediction of gene starts in microbial genomes. Implications for finding sequence motifs in regulatory regions. Nucleic Acids Res.

[40] Delcher A (1999). Improved microbial gene identification with GLIMMER. Nucleic Acids Res.

[41] Punta M, Coggill PC, Eberhardt RY, Mistry J, Tate J, Boursnell C (2012). The Pfam protein families database. Nucleic Acids Res.

[42] Quevillon E, Silventoinen V, Pillai S, Harte N, Mulder N, Apweiler R (2005). InterProScan: protein domains identifier. Nucleic Acids Res.

[43] Marchler-Bauer A, Lu S, Anderson JB, Chitsaz F, Derbyshire MK, DeWeese-Scott C (2011). CDD: a Conserved Domain Database for the functional annotation of proteins. Nucleic Acids Res.

[44] Krogh A, Larsson B, von Heijne G, Sonnhammer ELL (2001). Predicting transmembrane protein topology with a hidden markov model: application to complete genomes. J Mol Biol.

[45] Almagro Armenteros JJ, Tsirigos KD, Sønderby CK, Petersen TN, Winther O, Brunak S (2019). SignalP 5.0 improves signal peptide predictions using deep neural networks. Nat Biotechnol.

[46] Huang S, Wang K, Jiao N, Chen F (2012). Genome sequences of siphoviruses infecting marine *Synechococcus* unveil a diverse cyanophage group and extensive phage-host genetic exchanges. Environ Microbiol.

[47] Aziz RK, Bartels D, Best AA, DeJongh M, Disz T, Edwards RA (2008). The RAST Server: Rapid Annotations using Subsystems Technology. BMC Genomics.

[48] Richter M, Rosselló-Móra R, Oliver Glöckner F, Peplies J (2016). JSpeciesWS: a web server for prokaryotic species circumscription based on pairwise genome comparison. Bioinformatics.

[49] Arndt D, Grant JR, Marcu A, Sajed T, Pon A, Liang Y (2016). PHASTER: a better, faster version of the PHAST phage search tool. Nucleic Acids Res.

[50] Roberts RJ, Vincze T, Posfai J, Macelis D (2015). REBASE—a database for DNA restriction and modification: enzymes, genes and genomes. Nucleic Acids Res.

[51] Couvin D, Bernheim A, Toffano-Nioche C, Touchon M, Michalik J, Néron B (2018). CRISPRCasFinder, an update of CRISRFinder, includes a portable version, enhanced performance and integrates search for Cas proteins. Nucleic Acids Res.

[52] Tamura K, Stecher G, Kumar S (2021). MEGA11: molecular evolutionary genetics analysis version 11. Mol Biol Evol.

[53] Meier-Kolthoff JP, Göker M (2017). VICTOR: genome-based phylogeny and classification of prokaryotic viruses. Bioinformatics.

[54] Nakagawa S, Takai K, Inagaki F, Horikoshi K, Sako Y (2005). *Nitratiruptor tergarcus* gen. nov., sp. nov. and *Nitratifractor salsuginis* gen. nov., sp. nov., nitrate-reducing chemolithoautotrophs of the ε-*Proteobacteria* isolated from a deep-sea hydrothermal system in the Mid-Okinawa Trough. Int J Syst Evol Microbiol.

[55] Richter M, Rosselló-Móra R (2009). Shifting the genomic gold standard for the prokaryotic species definition. Proc Natl Acad Sci USA.

[56] Pedulla ML, Ford ME, Houtz JM, Karthikeyan T, Wadsworth C, Lewis JA (2003). Origins of highly mosaic mycobacteriophage genomes. Cell.

[57] Mercier C, Lossouarn J, Nesbø CL, Haverkamp THA, Baudoux AC, Jebbar M (2018). Two viruses, MCV1 and MCV2, which infect *Marinitoga* bacteria isolated from deep-sea hydrothermal vents: functional and genomic analysis. Environ Microbiol.

[58] Samson JE, Magadán AH, Sabri M, Moineau S (2013). Revenge of the phages: defeating bacterial defences. Nat Rev Microbiol.

[59] Meyer JL, Huber JA (2014). Strain-level genomic variation in natural populations of *Lebetimonas* from an erupting deep-sea volcano. ISME J.

[60] Frost LS, Leplae R, Summers AO, Toussaint A (2005). Mobile genetic elements: the agents of open source evolution. Nat Rev Microbiol.

[61] Ramisetty BCM, Sudhakari PA (2019). Bacterial ‘grounded’ prophages: hotspots for genetic renovation and innovation. Front Genet.

[62] Labrie SJ, Samson JE, Moineau S (2010). Bacteriophage resistance mechanisms. Nat Rev Microbiol.

[63] Piel D, Bruto M, Labreuche Y, Blanquart F, Goudenège D, Barcia-Cruz R (2022). Phage–host coevolution in natural populations. Nat Microbiol.

[64] Lynch KH, Stothard P, Dennis JJ (2010). Genomic analysis and relatedness of P2-like phages of the *Burkholderia cepacia* complex. BMC Genomics.

[65] Godde JS, Bickerton A (2006). The repetitive DNA elements called CRISPRs and their associated genes: evidence of horizontal transfer among prokaryotes. J Mol Evol.

[66] Nobrega FL, Walinga H, Dutilh BE, Brouns SJJ (2020). Prophages are associated with extensive CRISPR–Cas auto-immunity. Nucleic Acids Res.

[67] Yi H, Huang L, Yang B, Gomez J, Zhang H, Yin Y (2020). AcrFinder: genome mining anti-CRISPR operons in prokaryotes and their viruses. Nucleic Acids Res.

[68] Maier L-K, Lange SJ, Stoll B, Haas KA, Fischer SM, Fischer E (2013). Essential requirements for the detection and degradation of invaders by the *Haloferax volcanii* CRISPR/Cas system I-B. RNA Biol.

[69] Boudry P, Semenova E, Monot M, Datsenko KA, Lopatina A, Sekulovic O (2015). Function of the CRISPR-Cas system of the human pathogen *Clostridium difficile*. mBio.

[70] Barrangou R, Fremaux C, Deveau H, Richards M, Boyaval P, Moineau S (2007). CRISPR provides acquired resistance against viruses in prokaryotes. Science.

[71] Deveau H, Barrangou R, Garneau JE, Labonté J, Fremaux C, Boyaval P (2008). Phage response to CRISPR-encoded resistance in *Streptococcus thermophilus*. J Bacteriol.

[72] Nozawa T, Furukawa N, Aikawa C, Watanabe T, Haobam B, Kurokawa K (2011). CRISPR inhibition of prophage acquisition in *Streptococcus pyogenes*. PLoS ONE.

[73] Makarova KS, Wolf YI, Iranzo J, Shmakov SA, Alkhnbashi OS, Brouns SJJ (2020). Evolutionary classification of CRISPR–Cas systems: a burst of class 2 and derived variants. Nat Rev Microbiol.

