# Supplementary material for: Genomic insights into phage-host interaction in the deep-sea chemolithoautotrophic Campylobacterota, Nitratiruptor
Source: ISME Commun. 2022 Nov 1;2:108. doi: 10.1038/s43705-022-00194-5 (PMC9723563; doi:10.1038/s43705-022-00194-5)
Supplement: Supplementary file 10 — Supplemental Figures [file 43705_2022_194_MOESM10_ESM.pdf]

Supplementary information for the research article: Genomic insights into phage-host interaction in the deep-sea chemolithoautotrophic *Campylobacterota, Nitratiruptor*

Yukari Yoshida-Takashima[1]*, Yoshihiro Takaki[1], Mitsuhiro Yoshida[2], Yi Zhang[1], Takuro Nunoura[2], Ken Takai[1]

[1] Super-cutting-edge Grand and Advanced Research (SUGAR) Program, Institute for Extra-cutting-edge Science and Technology Avant-garde Research (X-star), Japan Agency for Marine-Earth Science and Technology (JAMSTEC), 2-15 Natsushima-cho, Yokosuka, Kanagawa 237-0061, Japan.
[2] Deep-Sea Bioresource Research Group, Research Center for Bioscience and Nanoscience (CeBN), Japan Agency for Marine-Earth Science and Technology (JAMSTEC), 2-15 Natsushima-cho, Yokosuka, Kanagawa 237-0061, Japan.

*Corresponding author: Yukari Yoshida-Takashima
E-mail: yukariyo@jamstec.go.jp
Address: 2-15 Natsushima-cho, Yokosuka, Kanagawa 237-0061, Japan.
Tel: +81-46-867-9690

SI Figures

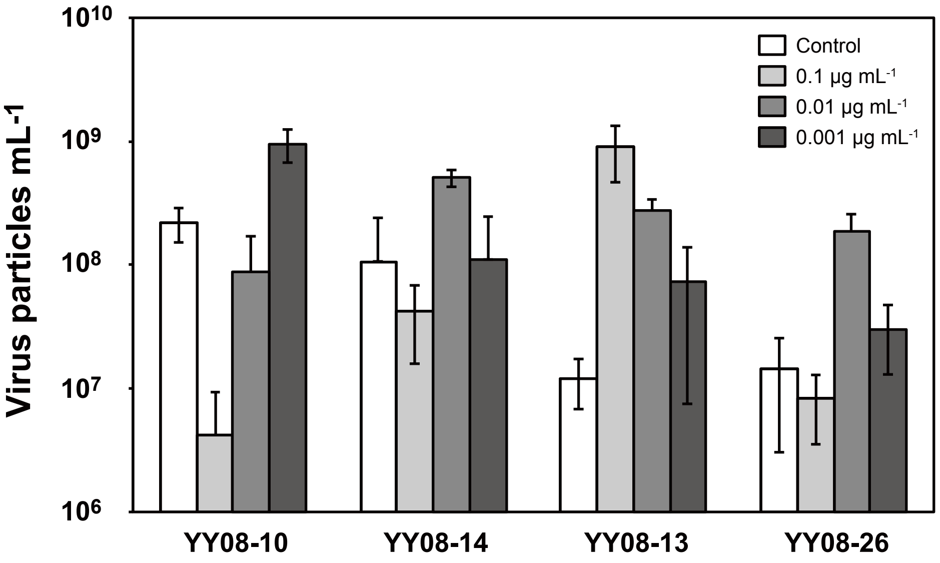

Fig. S1. Effect of prophage inductions by adding various concentrations of mitomycin C. Error bars show standard deviations (n=3).

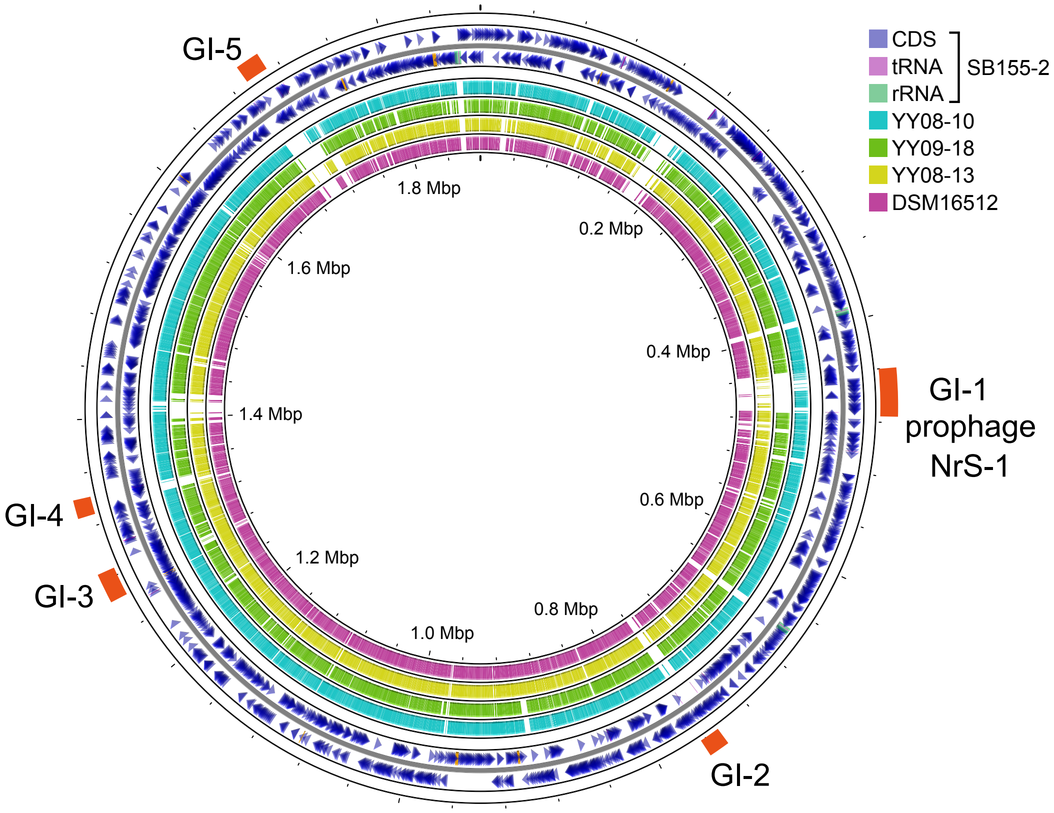

Fig. S2. Circular diagram of genomic sequence conservation of *Nitratiruptor* strains. A comparative genome map was drawn with the CGView Server [1]. *Nitratiruptor* sp. SB155-2 was used as the reference genome (outer two circles) and compared to a set of four other *Nitraiturptor* strains by BLASTP comparison (E-value of <10^{-5}). Orange labels denote genomic islands identified by IslandViewer4 [2], and GI-1 is consistent with temperate phage NrS-1.

References

1. Grant JR, Stothard P. The CGView Server: a comparative genomics tool for circular genomes. *Nucleic Acids Res* 2008; 36: W181–W184.
2. Bertelli C, Laird MR, Williams KP, Simon Fraser University Research Computing Group, Lau BY, Hoad G, et al. IslandViewer 4: expanded prediction of genomic islands for larger-scale datasets. *Nucleic Acids Res* 2017; 45: W30–W35.
